# Associations of Older Taiwanese Adults’ Personal Attributes and Perceptions of the Neighborhood Environment Concerning Walking for Recreation and Transportation

**DOI:** 10.3390/ijerph14121594

**Published:** 2017-12-18

**Authors:** Yung Liao, Pin-Hsuan Huang, Chih-Yu Hsiang, Jing-Huei Huang, Ming-Chun Hsueh, Jong-Hwan Park

**Affiliations:** 1 Department of Health Promotion and Health Education, National Taiwan Normal University, 162, Heping East Road Section 1, Taipei 106, Taiwan; liaoyung@ntnu.edu.tw (Y.L.); victorlove1610@gmail.com (P.-H.H.); vivianhsiang0301@gmail.com (C.-Y.H.); 2 School of Tourism and Hospitality Management, Temple University, 1801 N. Broad Street, Philadelphia, PA 19122, USA; jhuang32@ncsu.edu; 3 Department of Physical Education, National Taiwan Normal University, Taipei 106, Taiwan; boxeo19912016@gmail.com; 4 Institute of Convergence Bio-Health, Dong-A University, 32, Daeshingongwon-Ro, Seo-Gu, Busan 49201, Korea

**Keywords:** perceived environment, physical activity, Taiwan

## Abstract

This study examines the cross-sectional associations between personal and perceived neighborhood environment attributes regarding walking for recreation and transportation among older Taiwanese adults. Data related to personal factors, perceived environmental factors, and time spent engaging in transportation-related and recreational walking were obtained from 1032 older adults aged 65 years and above. The data were analyzed by carrying out an adjusted binary logistic regression. After adjusting for potential confounders, two commonly perceived environmental factors, the presence of sidewalks (PS) and the presence of a destination (PD), were positively associated with 150 min of walking for recreation. Different personal and perceived environmental factors were associated with walking for recreation and transportation. These findings suggest that policy-makers and physical activity intervention designers should develop both common and individual environmental strategies in order to improve and increase awareness of the neighborhood environment to promote recreational and transportation walking behaviors among older adults.

## 1. Introduction

Walking plays an important role in health promotion and disease prevention [1]. Walking is a low-cost and low-risk form of moderate-intensity aerobic physical activity that does not require special skills or facilities, and can be easily incorporated into the daily routine of individuals of all ages [2]. The health benefits of walking are greatest among older adults (aged 65+ years) because health problems related to inactivity are more common in this population [3]. However, as in many countries worldwide [4], most Taiwanese older adults do not attain the recommended levels of physical activity [5]. Walking is relatively easy to promote and adhere to [6,7], and thus has the potential to enable older adults to meet the recommendation for “a minimum of 30 min’ moderate-intensity physical activity” on most days in a week [8]. It is, therefore, critical to develop effective strategies that encourage older adults to walk, so as to meet their recommended levels of weekly physical activity.

From the perspective of an ecological model, manipulating environmental attributes can be expected to have a long-term impact on the walking habits of the general population [9]. A better understanding of the environmental factors associated with walking habits can inform urban design and planning initiatives. Perceptions of environmental attributes are particularly critical in the case of older adults, because the way in which these individuals interpret and make use of their neighborhood environments may affect their walking behaviors, especially given that older adults tend to spend more time in their own neighborhoods and to be sensitive to the state of that environment [10,11]. However, several of the perceived environmental factors that may influence older adults’ walking behaviors, such as aesthetic experiences, seeing active people, and a sense of traffic or crime safety, are difficult to measure objectively [10,12]. Moreover, because of social role transitions, older adults may have different walking patterns in different contexts (i.e., walking less to commute and more for recreation) than other age groups [13]. Thus, since age differences are associated with perceived environmental factors and walking [10], it is important to understand how perceived barriers and facilitators associated with the neighborhood walking behaviors of older adults can vary in different contexts. This information can enable policy-makers and physical activity intervention designers to develop effective strategies to promote walking among older adults.

Previous studies have examined the association between perceived environmental factors and walking for other purposes among older adults in the USA [14], Japan [13], Hong Kong [15,16], Belgium [17], and Brazil [18]. However, the relationship remains unclear in the context of other countries, such as Taiwan. Existing evidence has shown that the target group’s perception of neighborhood aesthetics [13,14,15], street connectivity, and the presence of good infrastructure [16] are consistently associated with walking for recreation; similarly, good street connectivity [14,19], lower crime, and traffic safety [16,17,18] are consistently associated with walking for transportation among older adults. Since Taiwan may have different cultural and environmental contexts than those previously studied, there is a need for context-specific data to inform policy-makers and physical activity intervention designers in Taiwan. This study, therefore, examines the association between perceived neighborhood attributes and walking for recreation and transportation among older Taiwanese adults.

## 2. Materials and Methods

### 2.1. Participants

In this study, data were collected in 2016 by administering a random-digit dialing, telephone-based, cross-sectional survey through a telephone research service company. In November 2016, Taiwan was estimated to have an older adult population of 3,089,843 (the target population) and an area of 36,192.8 km^2^. The sample size needed for the present study was estimated to be 1067 adults, given a 95% confidence level and 3% confidence interval. A stratified and clustered sampling process was used to select respondents. Trained interviewers administered a standardized questionnaire, after receiving two days of training. Household telephone numbers were selected at random by a computer-assisted system. At the beginning of each telephone interview, the interviewers confirmed whether or not there were any eligible respondents (older adults aged 65+ year) in the household. If there were, one respondent from each household was selected to participate. A total of 3546 older adults were asked to respond, and 1074 completed the survey (response rate: 30.3%). After data cleaning, 1032 participants provided complete data for our analysis (eligibility rate: 96.1%). No rewards were provided for participation. Verbal informed consent was obtained before the start of each telephone interview. The protocols and procedures of this study were reviewed and approved by the Research Ethics Committee of National Taiwan Normal University (REC number: 201605HM006).

### 2.2. Walking for Recreation and Transportation

Information on outcome variables, including transportation-related and leisure-time physical activities, was obtained using the Taiwanese version of the International Physical Activity Questionnaire (long version: IPAQ-LV) [20]. A telephone version of this survey has been developed in various languages and conducted in several countries [21]. This questionnaire exhibits high test–retest reliability (r = 0.78) and acceptable criterion validity (r = 0.31–0.41), compared with data obtained through accelerometers [20]. The second part of the IPAQ-LV was used to measure the frequency (number of days in the previous seven days) and duration (minutes per day) of engagement in “walking for transportation”. Time spent in leisure-time walking was measured using the fourth part of the IPAQ-LV. The total time spent engaging in leisure-time walking was determined by multiplying the frequency (per week) by the duration of leisure time (per day). As recommended by the IPAQ Scoring Protocol [21], the outcome variable was dichotomized because the distributions were skewed. In accordance with the Global Recommendations on Physical Activity for Health [2,8], the outcome variables were categorized into “achieving 150 min per week” and “not achieving 150 min per week”.

### 2.3. Perceived Environmental Variables

Perceived environmental attributes were identified using the Taiwanese version of the International Physical Activity Questionnaire-Environmental Module (IPAQ-E), which was translated using the World Health Organization’s process for translating and adapting instruments [22]. The details of IPAQ-E are described elsewhere [23]. In brief, the IPAQ-E questionnaire consists of 7 core, 4 recommended, and 6 optional items. In this study, 11 items related to walking were used to measure perceived environmental attributes, including (1) residential density, (2) access to shops, (3) access to public transportation, (4) presence of sidewalks, (5) access to recreational facilities, (6) crime safety at night, (7) traffic safety, (8) seeing people being active, (9) aesthetics, (10) connectivity of streets, and (11) presence of a destination. Items involving bicycles were not included in this study. These 11 items were measured using a four-point Likert scale (strongly disagree, somewhat disagree, somewhat agree, and strongly agree), apart from one question: “what is the main type of housing in your neighborhood”? For this question, there were five response options: detached single-family housing, apartment buildings with 2–3 stories, a mix of single-family housing and apartment buildings with 2–3 stories, condos with 4–12 stories, and condos with 13 stories or more [23]. Consistent with a previous study [24], “residential density” was divided into “detached single-family housing” and “other”. The other items were categorized as “agree” (strongly agree and somewhat agree) and “disagree” (somewhat disagree and strongly disagree).

### 2.4. Sociodemographic Variables

The sociodemographic variables included gender, age, occupational type, educational level, marital status, living status, residential area, self-rated health status, and body mass index (BMI). Age was divided into three categories: 65–74 years, 75–84 years, and 85+ years. The occupational types were categorized as “full-time job” and “no full-time job”. The educational levels included “non-tertiary degree” (less than 13 years) and “tertiary degree” (13 years or more). Marital status was classified as “married” or “unmarried” (including widowed, separated, and divorced). Living status was divided into “living with others” and “living alone”. The residential areas were categorized as “metropolitan” or “non-metropolitan”. There were two options for self-rated health status: “good” and “poor”. BMI was based on self-reported weight and height and grouped into two categories: “not overweight” (<24 kg/m^2^) and “overweight/obese” (≥24 kg/m^2^) according to data provided by the Health Promotion Administration of the Ministry of Health and Welfare [25].

### 2.5. Statistical Analyses

This study analyzed data from 1032 older Taiwanese adults who provided complete information about the relevant variables. A forced-entry adjusted logistic regression for gender, age, occupational type, educational level, marital status, living status, residential area, self-rated health status, and body mass index (BMI) was conducted to examine the association of 11 perceived environmental factors related to walking for recreation or transportation. Adjusted odds ratios (ORs) and 95% confidence intervals (CIs) were calculated for each variable. Inferential statistics were obtained using IBM SPSS Statistics for Windows, Version 23.0 (Armonk, NY, USA), and the level of significance was set at *p* < 0.05.

## 3. Results

### 3.1. Participant Characteristics

The respondents’ basic information is shown in Table 1. Their mean age was 72.3 ± 6.1 years. Of the respondents, 50.8% were male, 66.1% were 65–74 years old, 10.2% had full-time employment, 28.7% had a tertiary degree, 77.0% were married, 86.3% were living with others, 49.3% lived in metropolitan areas, 81.0% had a good self-rated health status, and 41.5% were overweight or obese. The proportion achieving 150 min/week of walking for recreational purposes was 72.2%, while 21.2% achieved 150 min/week of walking for transportation purposes.

### 3.2. Personal Factors Associated with Recreational Walking

The ORs for attaining 150 min/week of walking during leisure time are presented in Table 2 by gender, age, occupational type, educational level, marital status, living status, residential area, self-rated health status, and BMI. Table 2 shows that older adults without a full-time job (OR = 3.40; 95% CI: 2.15–5.35), and with a tertiary degree (OR = 1.64; 95% CI: 1.22–2.20) were more likely to achieve 150 min or more of walking as a leisure-time activity than their peers.

### 3.3. Personal Factors Associated with Walking for Transportation

Table 2 also shows that respondents living in metropolitan areas (OR = 1.98; 95% CI: 1.45–2.71) were more likely to engage in walking as a mode of transportation, compared with those who lived in non-metropolitan areas.

### 3.4. Perceived Environmental Factors Associated with Walking for Leisure

Table 3 shows that older adults who perceived their neighborhoods as having good access to shops (OR = 1.45; 95% CI: 1.04–2.03), sidewalks (OR = 1.50; 95% CI: 1.15–1.96), and recreational facilities (OR = 1.52; 95% CI: 1.12–2.06), and who saw people being active (OR = 1.52; 95% CI: 1.16–1.99), felt that their neighborhoods were aesthetically pleasing (OR = 1.31; 95% CI: 1.01–1.69), and walked towards destinations (OR = 1.56; 95% CI: 1.17–2.07) were more likely to walk 150 min/week for leisure than those who did not.

### 3.5. Perceived Environmental Factors Associated with Walking for Transportation

Table 3 also shows that older adults who whose neighborhoods had sidewalks (OR = 1.93; 95% CI: 1.37–2.72), and who had walked toward a destination (OR = 2.39; 95% CI: 1.60–3.58) were more likely to walk 150 min/week or more as a mode of transportation than those who did not. Older adults who felt that traffic made their neighborhoods less safe (OR = 0.72; 95% CI: 0.52–0.98) were less likely to walk 150 min/week as a mode of transportation than those who did not.

## 4. Discussion

This is one of few studies carried out in an Asian country to explore the association between personal and perceived environmental factors and walking for recreation or transportation in older adults. The most important finding of this study is that two commonly perceived environmental factors, the presence of sidewalks and that of a destination, were positively associated with walking for recreation and transportation. After adjusting for potential confounders, these two commonly perceived environmental factors helped older adults achieve 150 min/week. These results may reflect earlier findings that older adults who had access to good walking infrastructure and a set destination were more likely to engage in context-specific walking [10,16,17]. It may further reveal that improving and increasing the awareness of sidewalks and the importance of having a destination could be promising strategies for encouraging older adults to engage in health-enhancing levels of walking in both recreation and transportation related contexts.

One important finding was that different perceived environmental factors were associated with walking for recreation and transportation among older adults. Consistent with previous findings [10,13,14,15,16], older adults who perceived their neighborhoods as having easy to access to shops or low-cost recreational facilities (i.e., parks and recreation centers), active social environments (i.e., seeing other people being active), and positive aesthetic experiences (i.e., attractively landscaped, well-maintained, and clean environments) may be more motivated to engage in the recommended levels of recreational walking. Moreover, older adults who felt that traffic was making their neighborhoods unsafe were less likely to walk for transportation, a finding that has been consistently reported in previous studies [16,17,19]. This finding suggests that older adults are more sensitive to the state of the neighborhood environment [10,11] and to unsafe traffic (i.e., high vehicle speed, traffic volume, and accident rates). The perception of dangerous traffic could, therefore, be a key environmental barrier to transportation-related walking among older adults. The present findings may provide critical evidence, alerting policy-makers and physical activity intervention designers that, in addition to common strategies (increasing awareness of the presence of sidewalks and destinations), different intervention strategies should also be considered when promoting walking for recreation and transportation among older adults.

Another finding of this study was that older adults without full-time jobs and with higher levels of education were more likely to walk for recreation, while those living in non-metropolitan areas were less likely to walk for transportation purposes. Consistent with previous findings [16,17], older adults with lower educational levels may be more likely to have inactive lifestyles and lower health literacy [26,27], making them less likely to walk during their leisure time. In addition, the present results suggest that older adults without full-time jobs may have more spare time for recreational walking. Consistent with a previous study [17], older adults living in non-metropolitan areas were less likely to walk for transportation than those living in metropolitan areas. This result could be explained by the fact that non-metropolitan areas have low residential density, poor infrastructure, and poor access to public transportation. Thus, it is important to target older adults with lower levels of education and full-time jobs, encouraging them to engage in recreational walking. They should also be encouraged to improve and increase their awareness of infrastructure and transportation options in non-metropolitan areas in order to support walking for transportation among older adults.

This study has several limitations. First, because of the cross-sectional design, we could not draw conclusions regarding causal relationships. Secondly, the outcome and exposure variables of this study were self-reported, and thus subject to bias. Thirdly, the self-selection of neighborhoods and weather conditions (factors with the potential to confound our results) were not controlled in this study. Finally, this study could not obtain an entirely representative sample because it utilized a telephone-based survey method, which made it impossible to reach people who did not have access to a household telephone (estimated as being approximately 7.1% of the population in 2015) [28]. It was also impossible to contribute to a number of incomplete data. Thus, the findings of the present study may not be generalizable to the overall population. Despite these limitations, the strengths of the present study include its use of a nationwide sample of older adults and its ability to adjust for a number of potential confounders.

## 5. Conclusions

This study revealed that two commonly perceived environmental factors, the presence of sidewalks and a destination, were positively associated with walking during recreational and transportation-related contexts, ultimately helping older adults to achieve 150 min/week of walking. Different perceived environmental factors were also observed. These findings suggest that policy-makers and physical activity intervention designers should develop both common and specialized environmental strategies in order to improve and increase awareness of the neighborhood environment, as a way of promoting recreational and transportation-related walking behaviors among older adults.

## Figures and Tables

**Table 1:** Basic characteristics of all respondents (N = 1032).

Basic Characteristics	Study Sample		National Data ^a^
	N	%	
Mean age (SD)	72.3 (6.1)		
Gender			
Male	524	50.8%	46.3%
Female	508	49.2%	53.7%
Age (years)			
65–74	682	66.1%	56.8%
75–84	292	28.3%	31.7%
85+	58	5.6%	11.5%
Occupational type			
Full-time job	105	10.2%	8.5%
Non-full-time job	927	89.8%	91.5%
Educational			
Non-tertiary degree	736	71.3%	87.1%
Tertiary degree	296	28.7%	12.9%
Marital status			
Married	795	77.0%	60.9%
Unmarried	237	23.0%	39.1%
Living status			
Alone	141	13.7%	11.1%
With others	891	86.3%	88.9%
Residential area			
Non-metropolitan	523	50.7%	34.6%
Metropolitan	509	49.3%	65.4%
Self-rated health status			--- ^b^
Good	836	81.0%	---
Poor	196	19.0%	---
Body Mass Index (kg/m^2^)			--- ^b^
Non-overweight	604	58.5%	---
Overweight/obese	428	41.5%	---
Recreational walking			--- ^b^
<150 min/week	287	27.8%	---
150+ min/week	745	72.2%	---
Walking for transportation			--- ^b^
<150 min/week	813	78.8%	---
150+ min/week	219	21.2%	---

^a^ Data source: Department of Statistics, Ministry of Interior, Taiwan (2016), Ministry of Labor Republic of China, Taiwan (2016), Ministry of Health and Welfare, Taiwan (2013); ^b^ National data could not be found to compare with our data.

**Table 2:** Personal factors associated with walking for leisure and transportation.

Personal Factors	Leisure-Time Walking	Transportation Walking
	OR (95% CI)	OR (95% CI)
Gender		
Male	1.00	1.00
Female	0.79 (0.60–1.03)	1.20 (0.87–1.65)
Age (years)		
65–74	1.00	1.00
75–84	0.81 (0.61–1.08)	0.99 (0.70–1.39)
85+	0.61 (0.34–1.07)	0.92 (0.46–1.85)
Occupational type		
Full-time job	1.00	1.00
Non-full-time job	3.40 (2.15–5.35) **	1.10 (0.65–1.86)
Educational		
Non-tertiary degree	1.00	1.00
Tertiary degree	1.64 (1.22–2.20) **	0.92 (0.46–1.85)
Marital status		
Married	1.00	1.00
Unmarried	1.12 (0.79–1.59)	1.18 (0.78–1.78)
Living status		
Alone	1.00	1.00
With others	1.25 (0.82–1.91)	0.69 (0.42–1.13)
Residential area		
Non-metropolitan	1.00	1.00
Metropolitan	1.17 (0.91–1.51)	1.98 (1.45–2.71) **
Self-rated health status		
Good	1.00	1.00
Poor	0.80 (0.58–1.11)	0.81 (0.54–1.23)
Body mass index (kg/m^2^)		
Non-overweight	1.00	1.00
Overweight/obese	0.92 (0.71–1.19)	0.99 (0.73–1.35)

Adjusted for gender, age, occupational type, educational level, marital status, living status, residential area, self-rated health status, and body mass index; ** *p* < 0.001.

**Table 3:** Perceived environmental factors associated with walking for recreation and transportation.

Perceived Environmental Factors	N	%	Recreational Walking	Transportation-Related Walking
			OR (95% CI)	OR (95% CI)
Residential density				
High	937	90.8%	0.98 (0.63–1.52)	1.87 (0.97–3.61)
Low	95	9.2%	1.00	1.00
Access to shops				
Good	838	81.2%	1.45 (1.04–2.03) *	1.42 (0.91–2.21)
Poor	194	18.8%	1.00	1.00
Access to public transportation				
Good	836	81.0%	1.29 (0.92–1.82)	1.39 (0.89–2.18)
Poor	196	19.0%	1.00	1.00
Presence of sidewalks				
Yes	618	59.9%	1.50 (1.15–1.96) *	1.93 (1.37–2.72) **
No	414	40.1%	1.00	1.00
Access to recreational facilities				
Good	794	76.9%	1.52 (1.12–2.06) *	1.46 (0.98–2.18)
Poor	238	23.1%	1.00	1.00
Crime safety at night				
Not safe	174	16.9%	1.25 (0.89–1.75)	1.18 (0.77–1.79)
Safe	858	83.1%	1.00	1.00
Traffic safety				
Not safe	345	33.4%	0.98 (0.75–1.28)	0.72 (0.52–0.98) *
Safe	687	66.6%	1.00	1.00
Seeing people being active				
Yes	677	65.6%	1.52 (1.16–1.99) *	1.11 (0.80–1.55)
No	355	34.4%	1.00	1.00
Aesthetics				
Yes	562	54.5%	1.31 (1.01–1.69) *	1.06 (0.78–1.44)
No	470	45.5%	1.00	1.00
Connectivity of streets				
Good	671	65%	1.11 (0.85–1.44)	1.12 (0.81–1.56)
Poor	361	35%	1.00	1.00
Presence of destination				
Yes	726	70.3%	1.56 (1.17–2.07) *	2.39 (1.60–3.58) **
No	306	29.7%	1.00	1.00

Adjusted for gender, age, occupational type, educational level, marital status, living status, residential area, self-rated health status, and body mass index (BMI); * *p* < 0.05, ** *p* < 0.001.
